# Thrombospondin-4 as potential cerebrospinal fluid biomarker for therapy response in pediatric spinal muscular atrophy

**DOI:** 10.1007/s00415-024-12670-0

**Published:** 2024-09-06

**Authors:** Vera Dobelmann, Andreas Roos, Andreas Hentschel, Adela Della Marina, Markus Leo, Linda-Isabell Schmitt, Lorenzo Maggi, Ulrike Schara-Schmidt, Tim Hagenacker, Tobias Ruck, Heike Kölbel

**Affiliations:** 1 Department of Neurology, Medical Faculty and University Hospital Düsseldorf, Heinrich Heine University Düsseldorf, Moorenstr. 5, 40225 Duesseldorf, Germany; 2 Department of Pediatric Neurology, Developmental Neurology, and Social Pediatrics, Center for Neuromuscular Disorders in Children and Adolescents, Center for Translational Neuro- and Behavioral Sciences (C-TNBS), University Duisburg-Essen, Hufelandstr. 55, 45147 Essen, Germany; 3 Children’s Hospital of Eastern Ontario (CHEO) Research Institute, Ottawa, ON K1H 5B2 Canada; 4 Leibniz Institute of Analytical Sciences, ISAS, Dortmund, Germany; 5 Department of Neurology, Center for Translational Neuro- and Behavioral Sciences (C-TNBS), University Hospital Essen, Hufelandstr. 55, 45147 Essen, Germany; 6 Neuroimmunology and Neuromuscular Diseases Unit, Fondazione IRCCS Istituto Neurologico “Carlo Besta”, Milan, Italy

**Keywords:** Spinal muscular atrophy, Clinical proteomics, CSF biomarker, Thrombospondin-4, THBS4, TSP4

## Abstract

**Background and purpose:**

Spinal muscular atrophy (SMA) as the second most common neurodegenerative disorder in childhood is characterized by the deficiency of survival of motor neuron (*SMN*) protein leading predominantly to degeneration of alpha motor neurons and consequently to progressive muscle weakness and atrophy. Besides some biomarkers like *SMN2* copy number therapeutic biomarkers for SMA with known relevance for neuromuscular transmission are lacking. Here, we examined the potential of Thrombospondin-4 (TSP4) to serve as a cerebrospinal fluid (CSF) biomarker, which may also indicate treatment response.

**Methods:**

We used untargeted proteomic analyses to determine biomarkers in CSF samples derived from pediatric pre-symptomatic (*n* = 6) and symptomatic (*n* = 4) SMA patients. The identified biomarker TSP4 was then validated in additional 68 CSF samples (9 adult and 24 pediatric SMA patients, 5 adult and 13 pediatric non-disease controls in addition to 17 pediatric disease controls) by enzyme-linked immunosorbent assay (ELISA) as an additional analytical approach.

**Results:**

Untargeted proteomic analyses of CSF identified a dysregulation of TSP4 and revealed a difference between pre-symptomatic SMA patients and patients identified after the onset of first symptoms. Subsequent ELISA-analyses showed that TSP4 is decreased in pediatric but not adult SMA patients. CSF of pediatric patients with other neurological disorders demonstrated no alteration of TSP4 levels. Furthermore, CSF TSP4 levels of pediatric SMA patients increased after first dose of Nusinersen.

**Conclusions:**

We found that TSP4 levels are exclusively reduced in CSF of pediatric SMA patients and increase after treatment, leading us to the hypothesis that TSP4 could serve as a CSF biomarker with the potential to monitor treatment response in pediatric SMA patients. Moreover, TSP4 enable to distinguish pre-symptomatic and symptomatic patients suggesting a potential to serve as a stratification marker.

**Supplementary Information:**

The online version contains supplementary material available at 10.1007/s00415-024-12670-0.

## Introduction

With a prevalence of 1:10,000, 5q-associated spinal muscular atrophy (hereinafter SMA) is the second most common bi-allelic disease and the most common neurodegenerative disorder in childhood [1]. SMA is characterized by the detrimental reduction of survival of motor neuron (*SMN*) protein leading predominantly to degeneration of alpha motor neurons (MN) and consequently to progressive muscle weakness and atrophy [1]. SMA is mainly caused by a homozygous deletion on chromosome 5 within the *SMN1* gene [2]. The *survival* *motor* *neuron* *2* (*SMN2*) gene, which is a homologous copy of *SMN1*, is present in SMA in variable copies. Both genes encode the same protein, but *SMN2* lacks exon 7, which leads to an unstable SMNΔ7 protein [3]. Given that *SMN2* constantly produces approximately 10% functional SMN protein [4], *SMN2* copy number in the majority of patients inversely correlates with disease severity and onset and thus in the past stratifies for pre-symptomatic treatment [5]. Clinically, different SMA-phenotypes are defined based on the age of manifestation and clinical severity in type 0–4. Under now available therapies, the phenotypic spectrum is changing and now classified due to patients motor ability in “non-sitter”, “sitter”, and “walker”. Although MNs are the most affected cells in SMA, their loss may not exclusively depend on the absence of SMN: retrograde signals originating from skeletal muscles and neuromuscular junctions (NMJs) may also represent crucial players in MN vulnerability and contribute to the overall clinical manifestation [6]. Indeed, in mouse models of SMA, one of the earliest events detected is NMJ-defects [7] and clinically, walking performance can identify ambulant SMA patients with NMJ-dysfunction [8]. Moreover, a recent study demonstrated that SMN controls NMJ-integrity through U7 snRNP [9]. In the light of these facts, it is plausible that therapeutic intervention with Pyridostigmine, a quaternary carbamate mainly used to treat NMJ-disease by indirectly increasing the concentration of acetylcholine at the NMJ, showed beneficial outcomes in SMA patients [10, 11]; however, the primary endpoint showed no efficacy. Also, treatment with Salbutamol, a β-adrenergic agonist with an impact on NMJ, showed beneficial outcomes in SMA patients [12, 13], however, this may result from the known impact of Salbutamol on SMN protein stability and turnover [14–16]. Moreover, there is also an ongoing clinical trial with Amifampridine (3,4-Diaminopyridine) showing improvement in motor assessments [17].

Newly available therapies for SMA, specifically *SMN2* splicing modifiers such as Nusinersen or Risdiplam and gene replacement therapy (Onasemnogene Abeparvovec), have dramatically changed survival and overall disease progression [18, 19]. Nusinersen for instance is an antisense oligonucleotide, which corrects the splicing of *SMN2* pre-mRNA resulting in an increased production of functional SMN. An early initiation of treatment showed the strongest therapy response in SMA patients, but still the effect of this therapeutic intervention on motor functions varies between patients [20–24]. To improve the effectiveness and applicability of current therapies targeting SMN abundances, a profound understanding of the different pathophysiological aspects is important, especially with regard to the impact of varying residual endogenous SMN levels. Consequently, based on the definition of appropriate biomarkers, it could be helpful to obtain molecular information on whether therapies also result in more effective synaptic transmission at the NMJ and consequently reduce retrograde MN vulnerability.

Although *SMN2* copy number is valid as a biomarker of disease severity, alongside other infrequently used measures, it lacks a definitively reliable predictive value concerning ongoing disease activity. Particularly, it does not serve as an indicator of treatment response [25]. In the last years, neurofilaments (peripheral neurofilament light chain [pNF-L] and peripheral neurofilament heavy chain [pNF-H]) have been considered as biomarkers for SMA [26], but their clinical and analytical relevance is currently also being discussed [27]. Moreover, expression of the muscle-specific miRNA 206 has been linked to disease progression in a murine model of SMA [28]. However, therapeutic biomarkers with known relevance for neuromuscular transmission, for instance, based on known localizations and functional roles at the NMJ are still lacking.

Thrombospondin-4 (TSP4) is a member of the thrombospondin protein family, which represents a group of glycoproteins highly related to the extracellular matrix (ECM) [29]. Overall, thrombospondins participate in diverse biological functions, including cell adhesion and migration, cytoskeleton organization as well as cell–cell interactions and interactions between cells and the underlying matrix components [30–33]. The thrombospondin family consists of five members (TSP1-5), according to their functional domains the members can be classified into two subgroups: TSP1/2 and TSP3/4/5 [31]. Expression pattern of each thrombospondin differs in developing and adult tissue [31]. TSP4 is expressed in the developing embryo in *Xenopus*, however, in adult human tissue, it is mainly expressed in the heart and skeletal muscles [34]. Moreover, TSP4 has been identified as a factor contributing to neuropathic pain [35] and promoting neurite outgrowth [36], respectively. Along this line, results of previous studies highlighted an important role of TSP4 at NMJ in mice [36].

The present study aimed to identify new CSF biomarkers for SMA. To this end, in an exploratory proteomics-based analytical approach, protein signatures were compared in CSF samples derived from genetically proven pediatric SMA patients. Doing so, a comparison between patient with and without manifestation of clinical symptoms was carried out. These patients were identified by newborn screening for SMA in Germany [37]. This approach led to the identification of TSP4 discriminating between these two clinically diverging groups of pediatric patients. To validate our proteomic findings and to investigate the potential of TSP4 to serve as a therapy marker, we next analyzed CSF samples derived from SMA patients before and after Nusinersen therapy by enzyme-linked immunosorbent assay (ELISA).

## Patients, materials and methods

### Ethical considerations

All patients or their caregivers gave written informed consent. Study approval (for pediatric cases) was obtained from the University Duisburg-Essen ethics committee (approval number 19-9011-BO). Study approval (for adult cases) was also obtained from the University Duisburg-Essen ethics committee (approval number 18-8285-BO).

### Study design

To identify a new protein biomarker in CSF of SMA patients, a combined proteomic and ELISA-based analytical approach was applied in terms of an exploratory study: first, by making use of unbiased proteomic profiling in a data-dependent-acquisition mode, the protein signature of CSF derived from asymptomatic but genetically diagnosed pediatric SMA patients was compared to the one from symptomatic pediatric patients (discovery cohort). Out of the dysregulated proteins, one candidate was selected based on knowledge of the protein which may accord with the pathophysiology underlying in SMA in terms muscle denervation based on loss of motoneurons. Next, this promising biomarker candidate was validated in a larger cohort by making use of ELISA as an additional analytical approach which is applicable in standard laboratory settings. To this end, CSF from further pediatric SMA patients in addition to pediatric non-disease controls and pediatric disease controls (children suffering from other neurological conditions) was analyzed. The inclusion of pediatric disease controls aimed to address a potential specificity of the novel biomarker candidate. Based on the results obtained in the pediatric cohort, various aspects such as clinical SMA subtype, *SMN2* copy number and the therapeutic response to Nusinersen were taken into account to analyze the potential of our biomarker candidate so serve as a stratification and/or therapeutic marker. In addition, CSF samples derived from adult SMA patients and controls were included to investigate the potential of the protein to serve as a SMA biomarker in CSF also derived from adult patients. This approach is prompted by the fact that another SMA biomarker, LARGE1, was recently introduced as a Nusinersen-related therapy marker in adults but not in pediatric patients [38]. A more detailed description of the patients included in our study is provided in the paragraph below and a schematic representation of the study design is provided in supplementary Fig. 1.

### Patients and clinical data

#### SMA patients

For our proteomic-based discovery approach, CSF samples derived from a total of ten genetically confirmed pediatric SMA patients were included (six pre-symptomatic and four symptomatic cases; Suppl. Tab. 1). For ELISA-based quantification studies of TSP4 in terms of biomarker validation, CSF samples derived from 24 further pediatric cases with genetically proven SMA (pre-symptomatic, type 1, 2 or 3) as well as NDC were analyzed (Table 1). CSF samples were collected at different time points (baseline (V1), after 6 months (V2), after 12 months (V3) and after 24 months (V4). Control CSF (from NDC and disease controls) were collected by diagnostic procedures to test for central nervous system (CNS) diseases. Patients with therapeutic intervention (Nusinersen) were examined according to the Hammersmith Infant Neurological Examination Part 2 (HINE2) score, which is a simple to use neurological examination consisting of 26 items each scored on a scale of 0–3, designed for evaluating the motor milestones of children and already validated as a motor score in SMA-patients.

**Table 1:** Clinical data of included pediatric SMA patients

**#**	SMA subtype	*SMN2* copy number	Age of manifestation	Age at first dosing	Motor milestone at baseline (Visit 1)	Duration between disease manifestation and treatment	Visit 1 HINE2	Visit 2 HINE2	Visit 3 HINE2	Visit 4 HINE2
1	1	3	6 months	16 months	Non-sitter	10 months	16	18	18	21
2	Pre	4	N/A	4 months	Pre	N/A	9	24	26	R
3	Pre	2	N/A	3 weeks	Pre	N/A	1	Z	Z	Z
4	Pre	2	N/A	1 month	Pre	N/A	1	6	R	R
5	Pre	2	N/A	1 month	Pre	N/A	1	10	25	26
6	1	2	birth	3 weeks	Non-sitter	3 weeks	1	2	Z	Z
7	3	4	2 years	7 years	Walker	5 years	26	26	26	26
8	Pre	2	N/A	1 month	Pre	N/A	1	15	23	26
9	1	2	6 weeks	5 months	Non-sitter	3 months	2	N/A	N/A	N/A
10	Pre	3	N/A	1 month	Pre	N/A	2	15	24	26
11	3	4	2 years	4 years	Walker	2 years	24	24	24	26
12	Pre	3	N/A	3 weeks	Pre	N/A	1	18	N/A	N/A
13	3	4	2.5 years	4 years	Walker	1.5 years	26	26	26	26
14	Pre	3	N/A	1 month	Pre	N/A	1	13	23	26
15	3	3	2 years	3 years	Walker	1 year	25	26	26	26
16	2	3	6 months	3 years	Sitter	2.5 years	19	18	21	21
17	Pre	3	N/A	2 months	Pre	N/A	2	23	26	26
18	2	3	10 months	11 months	Sitter	1 month	19	13	17	17
19	2	3	10 months	11 months	Sitter	1 month	16	13	15	16
20	3	4	3 years	6 years	Walker	3 years	26	26	26	26
21	2	3	8 months	7 years	Sitter	6 years	10	9	9	9
22	2	2	6 months	8 years	Sitter	7.5 years	2	2	2	2
23	2	2	8 months	6 years	Sitter	5 years	8	7	9	10
24	1	2	2 months	6 months	Non-sitter	4 months	3	9	13	20

Moreover, eight CSF samples of genetically proven adult SMA patients (type 2 and 3) and five CSF samples of adult NDC were included in this study (Table 2). CSF samples derived from NDC were also collected by diagnostic procedures to test for CNS diseases. All adult SMA patients were treated with Nusinersen. Patients were examined using the Hammersmith Functional Motor Scale (HFMSE), which is a validated instrument with scoring values from 0 to 66 to assess the motor ability of children and adults with SMA type 2 and 3 with higher scores indicating better motor function.

**Table 2:** Clinical data of included adult SMA patients

#	SMA subtype	*SMN2* copy number	Age of manifestation (years)	Age at first dosing (years)	Motor milestone at baseline	Duration between disease manifestation and treatment (years)	Baseline HFMSE	2 months HFMSE	10 months HFMSE
1	3	4	23	41	Walker	18	53	58	45
2	2	3	1	33	Sitter	32	4	9	6
3	3	3	4	18	Walker	14	N/A	N/A	60
4	3	4	1.5	43	Walker	41.5	51	52	48
5	2	3	1	28	Sitter	27	3	6	6
6	3	5	16	46	Walker	30	36	42	42
7	3	4	3	29	Walker	26	57	59	61
8	3	4	39	61	Sitter	22	17	17	22

Age at first dosing refers to collection of baseline samples

### Proteomic analyses

Proteomic profiling on CSF samples derived from ten pediatric SMA cases (pre- and symptomatic) was carried out as described previously [39].

### Enzyme-linked immunosorbent assay (ELISA)

TSP4 levels were measured in CSF samples derived from SMA patients, various control diseases and NDC using the “Human TSP4 (Thrombospondin-4) ELISA Kit” (HUES02731, AssayGenie). The assay was performed according to the manufacturer’s protocol. In brief, CSF samples without further dilution were added to the TSP4-antibody-coated 96-well-plate and incubated for 90 min at 37 °C. Wells were then washed and the detection antibody was added, the plate was incubated for 60 min at 37 °C. Wells were washed again and buffer containing Horseradish peroxidase was added for 30 min at 37 °C. A further washing step followed and then the substrate solution was added for about 10 min at 37 °C, subsequently reaction was stopped using stopping solution. The optical density of each well was determined immediately after that using a microplate reader set at 450 nm.

## Results

### Characteristics of pediatric and adult SMA patients included in the study

In our discovery cohort, ten pediatric SMA patients with an age range between 3 weeks and 6.5 years at start of treatment were included: 40% (*n* = 4) of all patients had two *SMN2* copies, 50% (*n* = 5) had three *SMN2* copies and 10% (*n* = 1) had four *SMN2* copies. The six pre-symptomatic patients were identified in the context of the NBS for SMA. Two symptomatic patients were categorised as SMA type 1 and two as SMA type 3. In this sub-cohort, 60% (*n* = 6) of all patients were female and 40% (*n* = 4) were male (Suppl. Tab. 1).

In our pediatric validation cohort, 24 SMA patients were included with an age range between 3 weeks and 8 years at start of treatment: 33% (*n* = 8) had two *SMN2* copies, 42% (*n* = 10) had three *SMN2* copies, and 21% (*n* = 5) had four *SMN2* copies. Of all subjects, 46% (*n* = 11) were female and 54% (*n* = 13) were male. Here, eight patients were pre-symptomatic after diagnosis via NBS, five symptomatic patients were assigned to SMA type 1, six patients to SMA type 2 and five patients to SMA type 3. All clinically pre-symptomatic patients developed motor milestones in time, which was not expected in the natural disease course of SMA type 1 or 2. Clinical data of included SMA patients are summarized in Table 1. At the time of visit 3, four patients had already changed therapy to Risdiplam and two families have moved and were thus loss of follow-up.

In the adult validation cohort, eight patients between 29 and 61 years of age at start of treatment were included. Of these adult patients, 37.5% (*n* = 3) had three *SMN2* copies, 50% (*n* = 4) had four *SMN2* copies, and 12.5% (*n* = 1) had five *SMN2* copies. 50% (*n* = 4) of all patients were female and male, respectively. According to the current motor phenotype, 62.5% (*n* = 5) of all patients were classified as “walker” and 37.5% (*n* = 3) were classified as “sitter”. Clinical data of included adult SMA patients are summarized in Table 2.

### Thrombospondin-4 is altered in CSF of pediatric but not adult SMA patients

Untargeted proteomic profiling was performed on CSF samples derived from ten pediatric clinically discordant SMA patients: this discovery cohort included patients which already presented with symptoms (symptomatic; *n* = 4) as well as such diagnosed via NBS before developing first symptoms (pre-symptomatic; *n* = 6). A comparison of the proteomic signature between these two groups served to decipher protein markers of direct clinical relevance in SMA. This proteinogenic discovery approach unveiled the significant diverging abundance of nine proteins between the two patient groups (Suppl. Tab. 2, Suppl. Figure 2a). Among those proteins discriminating between pre- and symptomatic patients, TSP4 was selected as a promising functional candidate based on its profound dysregulation between the two patient groups (Suppl. Figure 2b) and its known localization to the NMJ [36]: pre-symptomatic SMA patients showed higher TSP4 CSF levels compared to the ones which already presented with clinical symptoms (Suppl. Figure 2b).

**Fig. 1:**
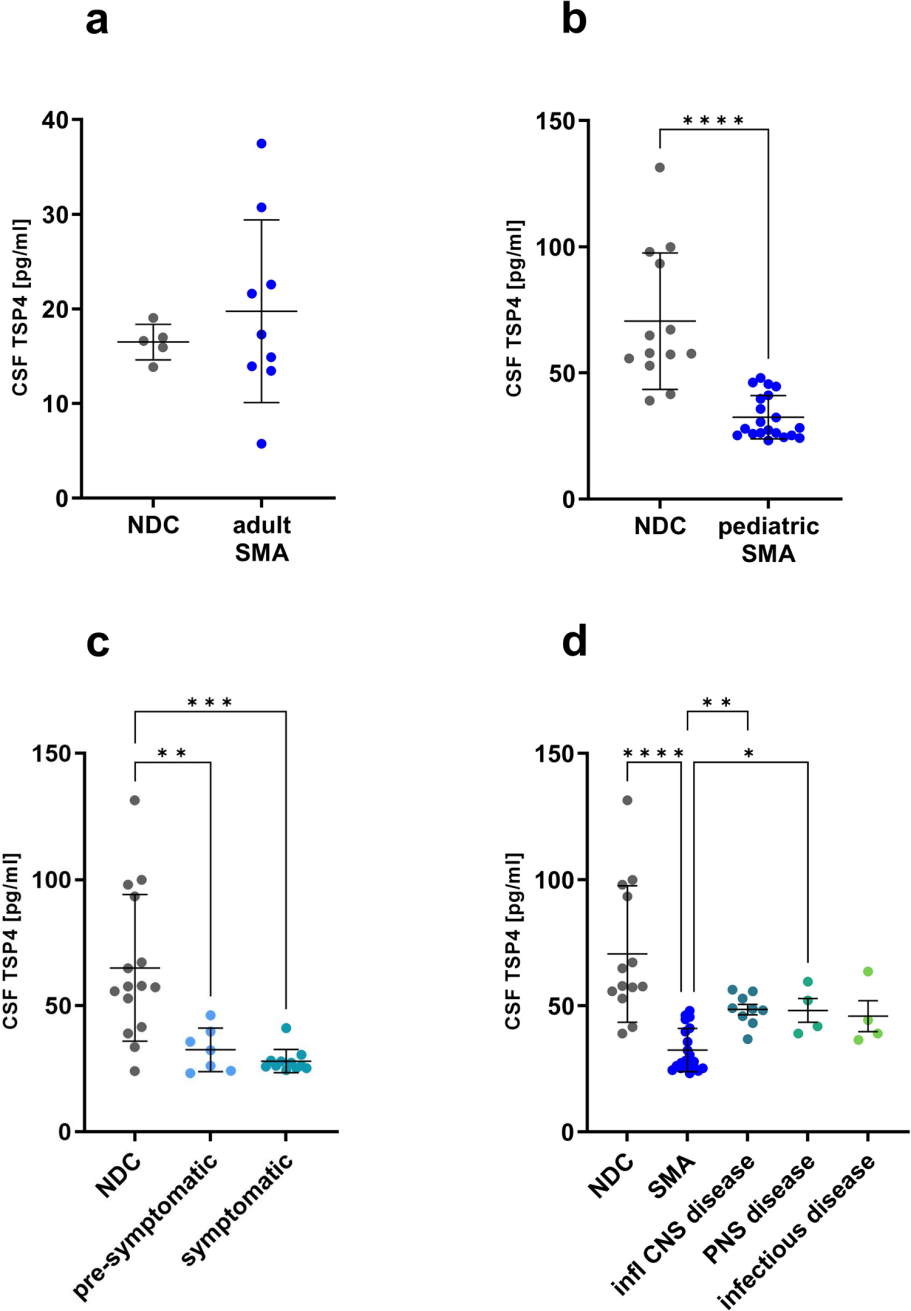
Thrombospondin-4 levels are altered in cerebrospinal fluid derived from pediatric but not adult SMA patients. **a** TSP4 levels in CSF derived from adult SMA patients (*n* = 9) and non-disease controls (NDC, *n* = 5) quantified via Enzyme-linked immunosorbent assay (ELISA). **b** TSP4 levels in CSF derived from pediatric SMA patients (*n* = 17) and NDC (*n* = 13) quantified via ELISA. **c** CSF TSP4 levels in pre- (*n* = 7) and symptomatically (*n* = 10) pediatric SMA patients and NDC (*n* = 13) quantified via ELISA. **d** CSF TSP4 levels in pediatric patients with SMA (*n* = 17), other inflammatory central nervous system (CNS) diseases (*n* = 11), peripheral nervous system (PNS) disorders (*n* = 4) and infectious diseases (*n* = 5). Data are represented as mean ± SD. Significance was tested by unpaired t test, Kruskal–Wallis or Mann–Whitney test, *p < 0.05, **p < 0.01, ***p < 0.001, ****p < 0.0001

To validate altered TSP4 CSF levels in further SMA patients, CSF derived from pediatric and adult SMA patients were analyzed via ELISA. Utilization of ELISA as an alternative quantification approach also aimed to validate our molecular findings by making use of a method which is more approachable in routine laboratory settings. In CSF derived from adult SMA patients, TSP4 was not altered compared to age-matched NDC (SMA: 19.74 ± 9.1, NDC: 16.49 ± 1.7, Fig. 1a). However, in the pediatric cohort, we identified significantly decreased CSF TSP4 levels compared to age-matched NDC (SMA: 29.99 ± 6.6, NDC: 70.52 ± 25.9 pg/ml, Fig. 1b). Notably, our ELISA-based quantification also revealed slightly higher TSP4 levels in pediatric patients identified pre-symptomatically compared to symptomatically, confirming the proteomic results (pre-symptomatic: 32.51 ± 7.9, symptomatic: 28.05 ± 4.4, NDC: 70.52 ± 25.9, Fig. 1c).

To investigate whether TSP4 represents a CSF biomarker specific for SMA in pediatric patients, we moreover analyzed CSF derived from pediatric patients suffering from other neurological diseases thus serving as disease controls. Compared to age-matched NDC, we detected no differences in the TSP4 levels of pediatric patients with inflammatory CNS diseases (multiple sclerosis), PNS diseases (peripheral fascial paresis) or infectious diseases (meningitis/encephalitis). Solely SMA patients showed significant decreased levels of TSP4 (SMA: 29.99 ± 6.6, NDC: 70.52 ± 25.9, inflammatory CNS disease: 46.51 ± 8.2, PNS disease: 48.13 ± 8.2, infectious disease: 45.85 ± 10.6 pg/ml, Fig. 1d).

**Fig. 2:**
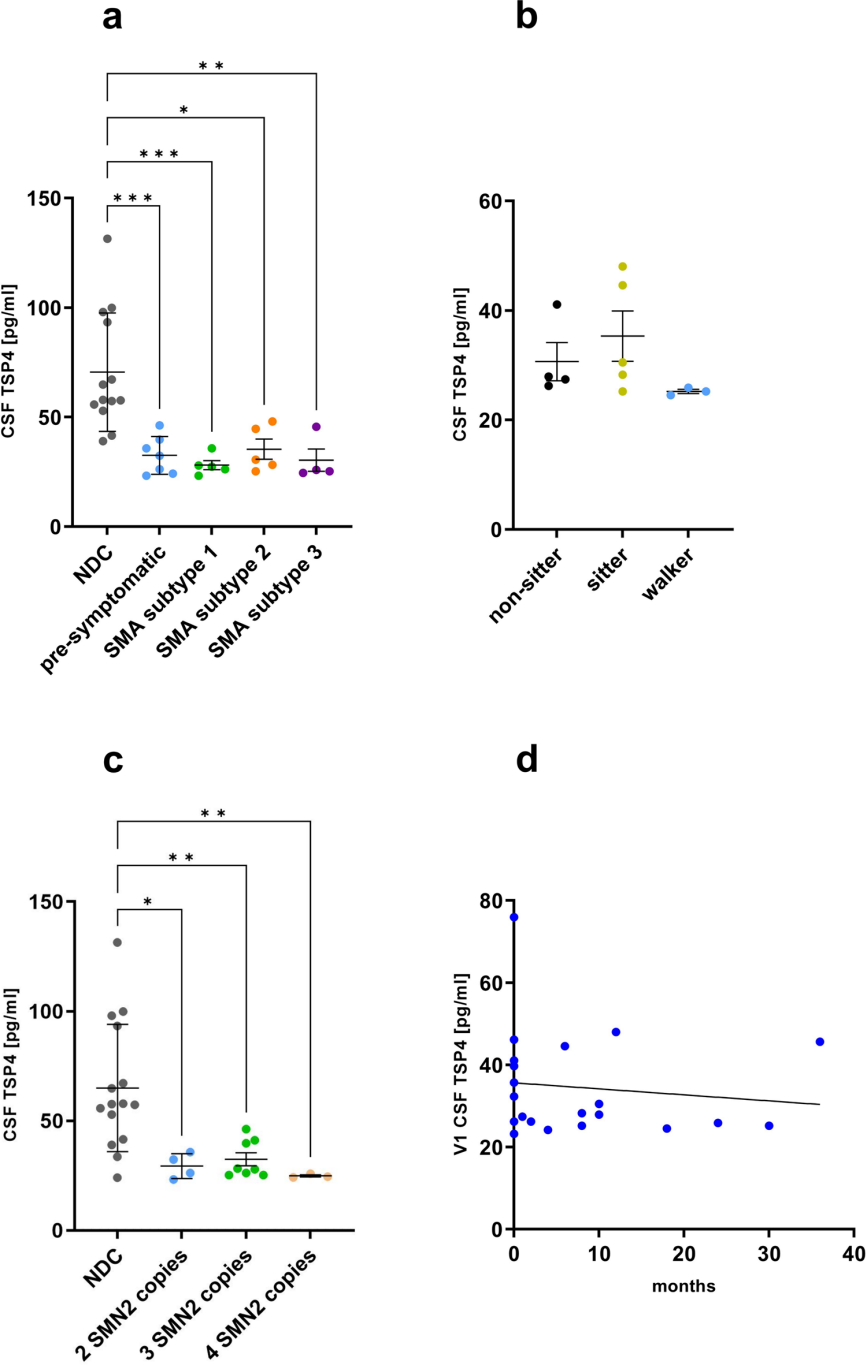
Thrombospondin-4 levels in cerebrospinal fluid derived from SMA patients do not differ between different clinical and genetic subtypes. **a** CSF TSP4 levels in pediatric pre-symptomatic patients (*n* = 7) and pediatric patients with SMA subtypes 1 (*n* = 4), 2 (*n* = 5) and 3 (*n* = 4). **b** TSP4 levels in pediatric SMA patients classified as non-sitter (*n* = 4), sitter (*n* = 5) and walker (*n* = 3) according to clinical data. **c** CSF TSP4 levels of pediatric patients with 2 (*n* = 4), 3 (*n* = 8) and 4 (*n* = 3) copies of the *SMN2* gene. **d** Correlation of TSP4 levels at initial sampling with age of disease onset. Data are represented as mean ± SD. Significance was tested by Kruskal–Wallis or Mann–Whitney test, *p < 0.05, **p < 0.01, ***p < 0.001

Further analyses of data obtained from the pediatric cohort showed no significant differences in TSP4 CSF levels regarding the respective SMA subtypes (NDC: 70.52 ± 25.9, pre-symptomatic: 32.51 ± 7.9, SMA type 1: 28.56 ± 4.5, SMA type 2: 27.9 ± 2.2, SMA type 3: 25.23 ± 0.6 or respectively non-sitter: 30.67 ± 6.1, sitter: 35.32 ± 9.2 and walker: 25.23 ± 0.6 pg/ml) or genotypes (according to the *SMN2* copy number; NDC: 70.52 ± 25.9, 2 *SMN2* copies: 29.36 ± 4.9, 3 *SMN2* copies: 32.48 ± 7.9, 4 *SMN2* copies: 24.89 ± 0.7 pg/ml) (Fig. 2a–c). In addition, TSP4 level at initial sampling did not correlate with age of disease onset (Fig. 2d).

To exclude an age-dependent effect of altered TSP4 level in CSF, we plotted concentrations against age of CSF sampling for pediatric and adult SMA patients, respective NDC as well as pediatric disease controls. Indeed, this approach showed no significant effect of age on TSP4 level in adult (Suppl. Figure 3a) or pediatric cohorts (Suppl. Figure 3b). Additionally, we also plotted the pediatric baseline CSF TSP4 levels against the time difference between age of first symptoms and start of treatment and we didn’t observe a significant effect (Suppl. Figure 3c).

In summary, our ELISA-findings displayed decreased TSP4 CSF levels solely for pediatric SMA patients independently from SMA subtype or *SMN2* copy number, adult SMA patients and pediatric patients with other neurological disorders showed no significant alteration compared to NDC.

### Thrombospondin-4 CSF levels increase under treatment of pediatric SMA patients with Nusinersen

**Fig. 3:**
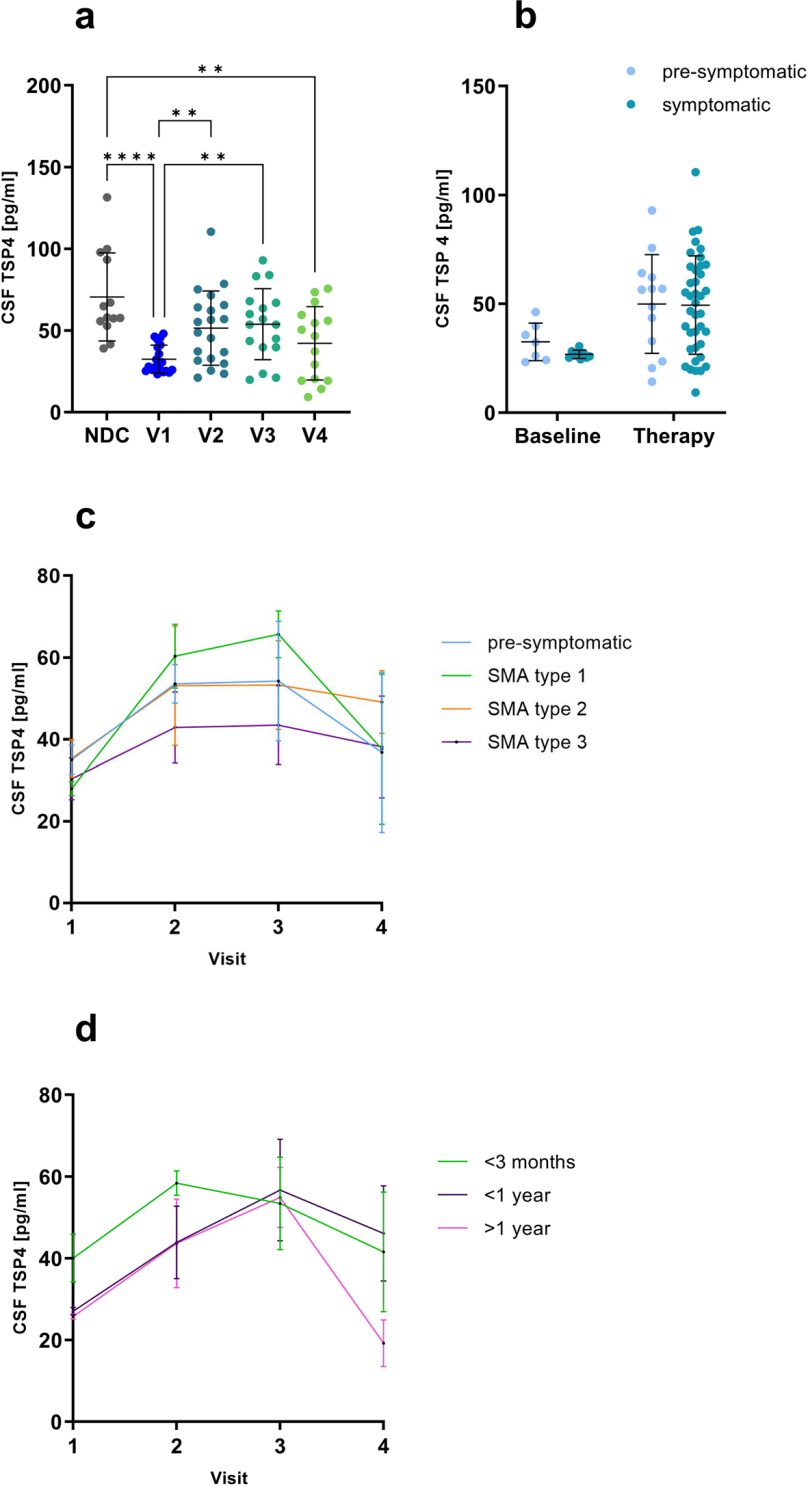
Thrombospondin-4 levels increase in cerebrospinal fluid derived from pediatric SMA patients under Nusinersen-treatment. **a** Thrombospondin-4 (TSP4) levels in cerebrospinal fluid (CSF) of pediatric patients with spinal muscular atrophy (SMA, *n* = 20) at four different time points (V1 = baseline, V2 = Ø 6 months, V3 = Ø 12 months, V4 = Ø 24 months) under treatment with Nusinersen. **b** Comparison of CSF TSP4 levels in pre- and symptomatically pediatric SMA patients at baseline and under Nusinersen-treatment. **c** Change of CSF TSP4 levels under Nusinersen-therapy for different SMA subtypes over time (V1 = baseline, V2 = Ø 6 months, V3 = Ø 12 months, V4 = Ø 24 months). **d** Change of CSF TSP4 levels under Nusinersen-therapy over time (V1 = baseline, V2 = Ø 6 months, V3 = Ø 12 months, V4 = Ø 24 months) grouped according to time of diagnosis. Data are represented as mean ± SD. Significance was tested by Kruskal–Wallis or Mann–Whitney test, *p < 0.05, **p < 0.01, ***p < 0.001, ****p < 0.0001

To investigate the potential of TSP4 to serve as a marker protein for therapy response in SMA, we next investigated whether TSP4 levels change in CSF under treatment with Nusinersen. Indeed, ELISA-based quantification studies unraveled significantly higher levels after 6 months (V2) of Nusinersen-treatment compared to baseline (V1). The following visits (V3, V4), reflecting a continuous 6 months treatment with Nusinersen, showed no further change of TSP4 in CSF derived from pediatric SMA patients under treatment (NDC: 70.52 ± 25.9, V1:32.41 ± 8.4, V2: 51.43 ± 22.1, V3: 53.82 ± 21.1, V4: 42.17 ± 21.7 pg/ml, Fig. 3a). In contrast to the baseline data, there was no longer any difference in TSP4 levels between pre-symptomatic and symptomatic patients during Nusinersen therapy (pre-symptomatic baseline: 32.51 ± 7.9, pre-symptomatic therapy: 49.89 ± 21.8, symptomatic baseline: 26.81 ± 1.8, symptomatic therapy: 49.42 ± 22.3 pg/ml, Fig. 3b).

We next examined CSF TSP4 levels under Nusinersen therapy for the individual SMA-subtypes: overall, subtypes show a similar elevation, with SMA subtype 1 showing the most pronounced increase and highest values after 12 months (V3) and the most pronounced decrease after 24 months (V4) (SMA1 V1: 28.09 ± 4.1, V2: 58.07 ± 14.9, V3: 64.51 ± 12.4, V4: 37.59 ± 18.4 pg/ml). SMA subtype 3 showed the least change and the lowest TSP4 levels along all visits (SMA3 V1: 30.33 ± 8.8, V2: 42.87 ± 17.4, V3: 43.42 ± 16.7, V4: 38.14 ± 21.5 pg/ml). Pre-symptomatic patients and patients with SMA type 2 showed an almost identical elevation over all visits with a difference at visit 4, where pre-symptomatic patients showed slightly lower levels than patients with SMA type 2 (pre-symptomatic V1: 34.96 ± 8.0, V2: 53.57 ± 10.5, V3: 54.24 ± 25.3, V4: 36.78 ± 27.6, SMA2 V1: 35.32 ± 9.2, V2: 53.11 ± 32.6, V3: 53.25 ± 21.67, V4: 49.08 ± 17.2 pg/ml) (Fig. 3c). Patients treated within 3 months after birth showed higher baseline TSP4 levels (40.05 ± 15.3 pg/ml) than patients diagnosed within the first year (27.06 ± 2.1 pg/ml) or after 1 year (25.77 ± 1.1 pg/ml). Patients diagnosed within 3 months showed an increase of TSP4 levels upon therapeutic intervention at visit 2 (58.38 ± 6.0 pg/ml), followed by a decrease over the next visits (V3: 53.44 ± 22.6, V4: 41.58 ± 25.3 pg/ml). Patients diagnosed within or after the first year showed an increase of TSP4 levels at visit 2 (< 1 year: 43.88 ± 19.8, > 1 year: 43.65 ± 18.8 pg/ml) and 3 (< 1 year: 56.69 ± 24.9, > 1 year: 54.91 ± 12.7 pg/ml), followed by a (strong) decrease at visit 4 with patients diagnosed after 1 year showing the lowest TSP4 levels (< 1 year: 46.12 ± 20.2, > 1 year: 19.21 ± 8.1 pg/ml) (Fig. 3d).

Although we identified an increase of TSP4 in the CSF under treatment with Nusinersen indicating the potential of TSP4 to serve as a therapeutic biomarker, there was no correlation between TSP4 increase (as a molecular response to therapeutic intervention) and improvement of clinical parameters, such as HINE2 (Suppl. Figure 4).

## Discussion

In this study, we utilized proteomic profiling aiming to unveil clinically relevant biomarkers in CSF derived from pediatric SMA patients. Our unbiased data-dependent acquisition approach identified nine proteins with significant diverging abundances including TSP4 as a protein presenting with altered levels between symptomatic and non-symptomatic therapy naïve children. Further confirmatory studies utilizing ELISA as an independent quantification approach show that TSP4 levels in CSF are reduced in therapy naïve pediatric SMA patients but not in adult patients. The robust quantification of TSP4 by ELISA highlights that this protein can also be studied by making use of analytical approaches, which are easily accessible in routine diagnostic settings, an important aspect regarding the need of easy-approachable patient stratification methods. Results of ELISA-based quantification studies moreover highlighted that the significant TSP4 decrease in CSF is not only present by the comparison of pediatric SMA patients with age-matched NDC but also by comparing these patients with pediatric patients suffering from other neurological diseases. Although this suggests a specificity of TSP4 as a CSF biomarker in children suffering from SMA, further studies on extended cohorts are needed to prove this hypothesis. With this requirement, however, it should be kept in mind that the occurrence of other MN diseases in childhood, especially in the first years of life, is extremely rare, making it difficult to investigate large cohorts of diverse genetic entities.

Further studies focusing on the effect of Nusinersen in the therapeutic intervention of pediatric SMA patients revealed an elevation of TSP4 CSF level upon treatment. This finding indicates that TSP4 may hold the potential to serve as a therapy marker for pediatric patients with SMA. Nusinersen is a non-systemic therapy that increases the expression of stable and functional SMN and thus strengthens the MN, which in turn is able to increasingly innervate the muscle again. A crucial process for the reinnervation is the “rebuilding” of synaptic sites [40]. TSP4 is an ECM protein and ECM components are known to be involved in neurite outgrowth, axonal pathfinding, and synapse formation [36]. In adult nervous system, TSP4 is expressed in certain neuronal populations and accumulates at the NMJ and at certain synapse-rich layers [36]. Of note, TSP4 has been localized to the NMJ also in mice and a protective role within the CNS was demonstrated [40, 41].

Thus, increased levels in clinically pre-symptomatic compared to symptomatic patients and TSP4 increase in CSF upon treatment might either reflect stage MN-damage associated with impaired release of secretory proteins based on apoptosis and MN repair/rescue based on Nusinersen treatment or elevated expression and thus secretion in terms of a rescue mechanism activated in the pre-symptomatic patients. However, further functional studies on animal models (therapy naïve versus treated) are needed to address the exact role of TSP4 in the molecular etiology of SMA. Nonetheless, our data combined with knowledge from literature suggest that TSP4—as a matricellular glycoprotein—may be involved not only in proper function of the CNS but also of the NMJs as a known main pathophysiological target of SMA [7, 8] with therapeutic relevance [10, 11]. Our results also support this assumption by the fact that decreased TSP4 levels in CSF elevate after pediatric patients were treated with Nusinersen which is targeting the expression of a stable SMN protein, improving MN function and thus restoring proper neuromuscular transmission. Consequently, we postulate that TSP4 levels in CSF may serve as a promising therapeutic biomarker of pathophysiological relevance in children suffering from SMA and hereby even enable to distinguish between clinically affected and non-affected patients in terms of a therapy marker.

Although, results of our quantification studies clearly indicate the potential of TSP4 to serve as a therapy marker, altered/restored levels in CSF upon treatment did not correlate with clinical outcome measures. Along this line, a biomarker study on 16 adults with SMA type 3 and 4 under Nusinersen treatment over 22 months reported on a significant decrease of pNF-H in CSF. However, this decrease was also not correlating with clinical outcome measures and a similar finding was obtained for Chitotriosidase-1 (CHIT1) levels. In contrast, a decrease of Chitinase-3-like protein 1 (YKL-40) strongly correlated with improvements in the revised upper limb module (RULM) [42]. These findings indicate that therapy markers in SMA do not necessarily correlate with clinical outcome measures even in adult patients in which—in contrast to children (see below “limitations of the study” section)—uniformed standardized motor tests such as RULM can be applied. However, it has to be taken into consideration that SMA belongs to the group of rare diseases and thus that significance in correlations might be limited by the number of patient-derived samples available for these studies.

Further studies on pediatric cases treated with other SMN-targeting drugs (such as Salbutamol [14–16]) would be needed to draw final conclusions regarding the potential of TSP4 to serve as a generalized therapy marker. Moreover, more detailed biochemical studies are needed to decipher the exact molecular background of TSP4 increase upon restoration of appropriate SMN expression. Based on the functional role of TSP4 at synapses [35] including NMJs [36], its potential to serve as a disease relevant biomarker in SMA—even enabling patient stratification in terms of distinguishing between clinically non-affected and affected patients as well as type 1 therapy responders versus type 2 and 3 responders—is underlined by its pathophysiological meaning. Taking the informative potential of TSP4 only in pediatric but not adult SMA-patients into consideration, one might speculate that this molecular observation is based on a development-dependent expression of the protein.

### Limitations of the study

Due to the heterogeneity of the patients with regard to the onset of the disease, the different time points of the treatment initiation and the severity of the disease, detailed analyses of the motor development of the children in relation to the TSP4 level could not be carried out. Based on the wide age range of our pediatric patient group (infant to school-age child), we were unable to use a uniform standardized motor test for this group apart from the HINE2. Furthermore, four families also changed therapy at their own request. Reasons for this decision were manifold and were notably not related to a lack of response to the therapy with Nusinersen. Larger and more homogeneous patient groups are needed to further validate TSP4 as an adequate biomarker in pediatric SMA. Based on the limitation of CSF available, CSF samples derived from children included in the proteomics-based discovery study could not be included in the ELISA-based quantification of TSP4. The assumed pathophysiological relevance of TSP4 in the molecular etiology of SMA discussed in this study is only hypothetical based on literature and additional functional studies are doubtless crucial to provide final evidence.

The most promise of TSP4 as a novel SMA biomarker may be for countries for which a treatment of patients with four *SMN2* copies is currently not reimbursed making use of TSP4 to monitor disease onset. However, in this case, TSP4 should ideally be measurable in minimal-invasive manner.

## Supplementary Information

Below is the link to the electronic supplementary material.

Schematic overview of samples included in the study along with applied analytical approaches (TIFF 135963 KB)

Proteomic findings unveiling altered Thrombospondin-4 levels in cerebrospinal fluid derived from pediatric SMA patients (a) Heatmap showing general differences between proteins quantified in cerebrospinal fluid (CSF) derived from pre-symptomatic (n=6) and symptomatic (n=4) identified pediatric patients with spinal muscular atrophy (SMA). (b) Thrombospondin-4 (TSP4) levels in CSF of pre- (n=6) and symptomatic (n=4) pediatric SMA patients (TIFF 135963 KB)

Correlation of Thrombospondin-4 levels in cerebrospinal fluid derived adult and pediatric SMA patients with age of sampling (a) Correlation of cerebrospinal fluid (CSF) Thrombospondin-4 (TSP4) levels with age of CSF sampling in adult spinal muscular atrophy (SMA) patients as well as adult non-disease controls (NDC). (b) Correlation of CSF TSP4 levels with age of CSF sampling in pediatric SMA patients, pediatric NDCs and pediatric patients with other neurological disorders (c) Correlation of pediatric baseline CSF TSP4 levels with difference between time of first symptoms and time of first treatment (TIFF 135963 KB)

Correlation of Thrombospondin-4 levels in cerebrospinal fluid derived from treated pediatric SMA patients with HINE2 score (a) Correlation of cerebrospinal fluid (CSF) Thrombospondin-4 (TSP4) difference between visit 1 and visit 3 under Nusinersen therapy with HINE2 score difference between visit 1 and visit 3 (b) Correlation of CSF TSP4 difference between visit 1 and visit 4 under Nusinersen therapy with HINE2 score difference between visit 1 and visit 3 (TIFF 135963 KB)

Supplementary file5 (DOCX 21 KB)

## Data Availability

The data that support the findings of this study are available on proper request from the corresponding author.
