# Supplementary material for: Thrombospondin-4 as potential cerebrospinal fluid biomarker for therapy response in pediatric spinal muscular atrophy
Source: J Neurol. 2024 Sep 6;271(10):7000–11. doi: 10.1007/s00415-024-12670-0 (PMC11446971; doi:10.1007/s00415-024-12670-0)
Supplement: Supplementary file 5 — Supplementary file5 (DOCX 21 KB) [file 415_2024_12670_MOESM5_ESM.docx]

**Supplementary figures and tables**

**Supplementary Table 1 Clinical data of pediatric patients included in the discovery cohort** #=patient ID; f=female; m=male; y=years; m=months; w=weeks; pre=pre-symptomatic.

| **#** | **SMA subtype** | ***SMN2* copy number** | **Motor milestone at baseline (visit 1)** |
| --- | --- | --- | --- |
| 1 | pre | 3 | pre |
| 2 | pre | 2 | pre |
| 3 | pre | 3 | pre |
| 4 | pre | 3 | pre |
| 5 | pre | 2 | pre |
| 6 | pre | 2 | pre |
| 7 | 3 | 4 | walker |
| 8 | 1 | 2 | non-sitter |
| 9 | 1 | 3 | non-sitter |
| 10 | 3 | 3 | walker |

**Supplementary Table 2 Protein with differential abundance between symptomatic and pre-symptomatic SMA patients**

| **Protein name** | **Ratio in symptomatic patients** | **p-value** |
| --- | --- | --- |
| Apolipoprotein F (APOF) | 1.98 | 0.046 |
| Inter-alpha-trypsin inhibitor heavy chain H4 (ITIH4) | 1.53 | 0.003 |
| Mannan-binding lectin serine protease 1 (MASP1) | 0.70 | 0.010 |
| Adiponectin (ADIPO) | 0.58 | 0.053 |
| Coagulation factor X (FA10) | 0.55 | 0.049 |
| Coagulation factor XIII B chain (F13B) | 0.52 | 0.038 |
| von Willebrand factor (VWF) | 0.41 | 0.011 |
| Peptidase inhibitor 16 (PI16) | 0.38 | 0.016 |
| Thrombospondin-4 (TSP4) | 0.35 | 0.016 |
